# Spontaneous Recycling of Electrosprayed Sample by Retrograde Motion of Microdroplets

**DOI:** 10.1021/jasms.3c00444

**Published:** 2024-02-14

**Authors:** Ochir Ochirov, Pawel L. Urban

**Affiliations:** Department of Chemistry, National Tsing Hua University, 101, Section 2, Kuang-Fu Rd., Hsinchu 300044, Taiwan

## Abstract

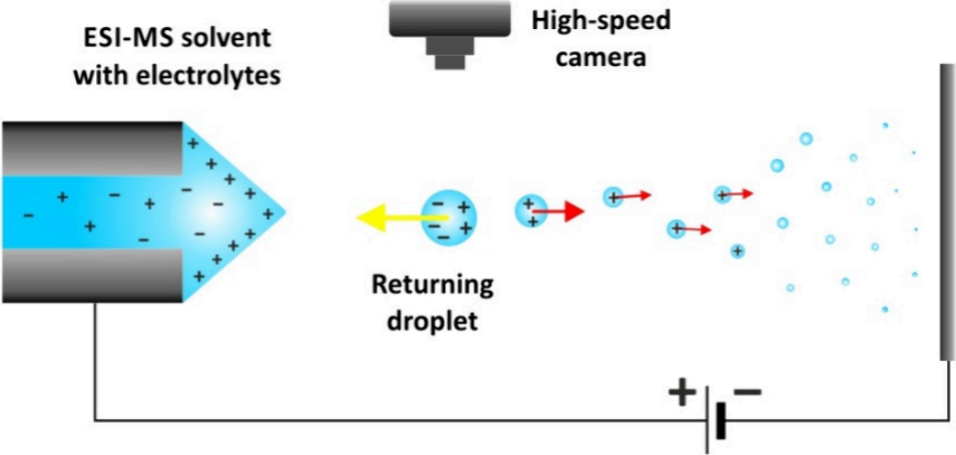

Here, we discuss an interesting phenomenon occurring spontaneously near the sample liquid meniscus at the tip of the electrospray emitter. While most ejected droplets move from the emitter tip toward the counter electrode, some of the droplets decelerate and move backward to the liquid meniscus. When they hit the surface of the liquid meniscus, they either merge with the bulk liquid or get recharged during intermittent contact with the liquid meniscus and immediately reaccelerate toward the counter electrode. In some cases, while in contact with the meniscus they spontaneously form a secondary Taylor cone and emit progeny droplets. This observation suggests that the amount of electric charge transferred to such a droplet is sufficient to surpass the Rayleigh limit. Similar effects were previously observed for water as well as for NaCl–water and ethanol–water mixtures. However, here we observed it for electrolyte solutions commonly used in electrospray ionization mass spectrometry: methanol–water solutions with the addition of ammonium acetate, formic acid, or ammonium hydroxide. The reported phenomenon reveals the ongoing recycling of sample liquid in electrosprays. Such recycling can contribute to enhancement of sample utilization efficiency in electrospray ionization.

## Introduction

Electrospray ionization (ESI) stands out as one of the most prevalent and advantageous techniques for ionization in mass spectrometry (MS).^1−3^ Apart from the growing pool of applications of ESI-MS, in the past three decades, there has been much interest in clarifying the mechanism of this technique.^4−7^ However, the mechanism is highly complex. Some studies investigated the dynamics of the liquid meniscus (Taylor cone) and droplet cloud (plume).^8−12^

Pulsation phenomena in ESI have been studied for more than two decades now. For example, Juraschek and Röllgen conducted measurements of capillary current while observing the disintegration of the liquid at the capillary tip with optical microscopy used in conjunction with a flash lamp.^13^ Marginean et al. showed that Taylor cone deformations play a central role in the mechanism of electrostatic spraying.^14^ They demonstrated that there exist four phases of the cone pulsation cycle: liquid accumulation, cone formation, emission of a jet, and relaxation. The authors provided evidence linking spray current oscillations to Taylor cone pulsation.^14^ The same group employed stroboscopic shadowgraphy with a laser source to explore the changes in ion production that accompany the transitions among the burst mode, the pulsating Taylor cone mode, and the cone-jet mode of electrospray.^15^ The primary droplets were produced by varicose waves and lateral kink instabilities on the liquid jet emitted from a Taylor cone, while secondary droplets were formed by fission.^15^ According to Gomez and Tang, the velocities of electrospray droplets, measured by phase Doppler anemometry, reach the values of several meters per second, both negative and positive.^16^

High-speed imaging enables tracking electrospray droplets.^17,18^ For example, Kim et al. implemented a high-speed camera to study the development of electrospray.^19^ They obtained visual information on how electrospray initiates, develops, and produces droplets of different sizes.

In 2011, Agostinho et al. reported on the retrograde motion and coalescence of deionized water microdroplets in electrospray captured by a high-speed camera.^20^ In addition to water, they also investigated NaCl–water and ethanol–water mixtures. However, we felt it necessary to investigate whether the same phenomenon occurs under the conditions of ESI-MS. Thus, we have repeated the previously described experiment using typical electrolytes used in ESI-MS. Similar to some of the reports cited above, in the present study, we utilized a high-speed camera to record the dynamics of droplets detaching from the Taylor cone.

## Experimental Section

### Materials

Ammonium acetate (≥98% purity, for HPLC, acetic acid ammonium salt) and formic acid (≥98% purity) were purchased from Acros Organics (Geel, Belgium). Methanol (for LC) was purchased from Merck (Darmstadt, Germany). Ammonium hydroxide solution (30–33% in water, w/w) was purchased from Sigma-Aldrich (St. Louis, MO, USA). Ammonium acetate (10 mM) and formic acid (0.5%, v/v) were prepared in 25% (v/v) methanol in deionized water. 225 μL of ammonium hydroxide solution was mixed with 29.8 mL of 25% (v/v) methanol in deionized water in order to achieve an approximate volumetric concentration of 0.5% ammonium hydroxide.

### Electrospray Setup

**Figure 1:**
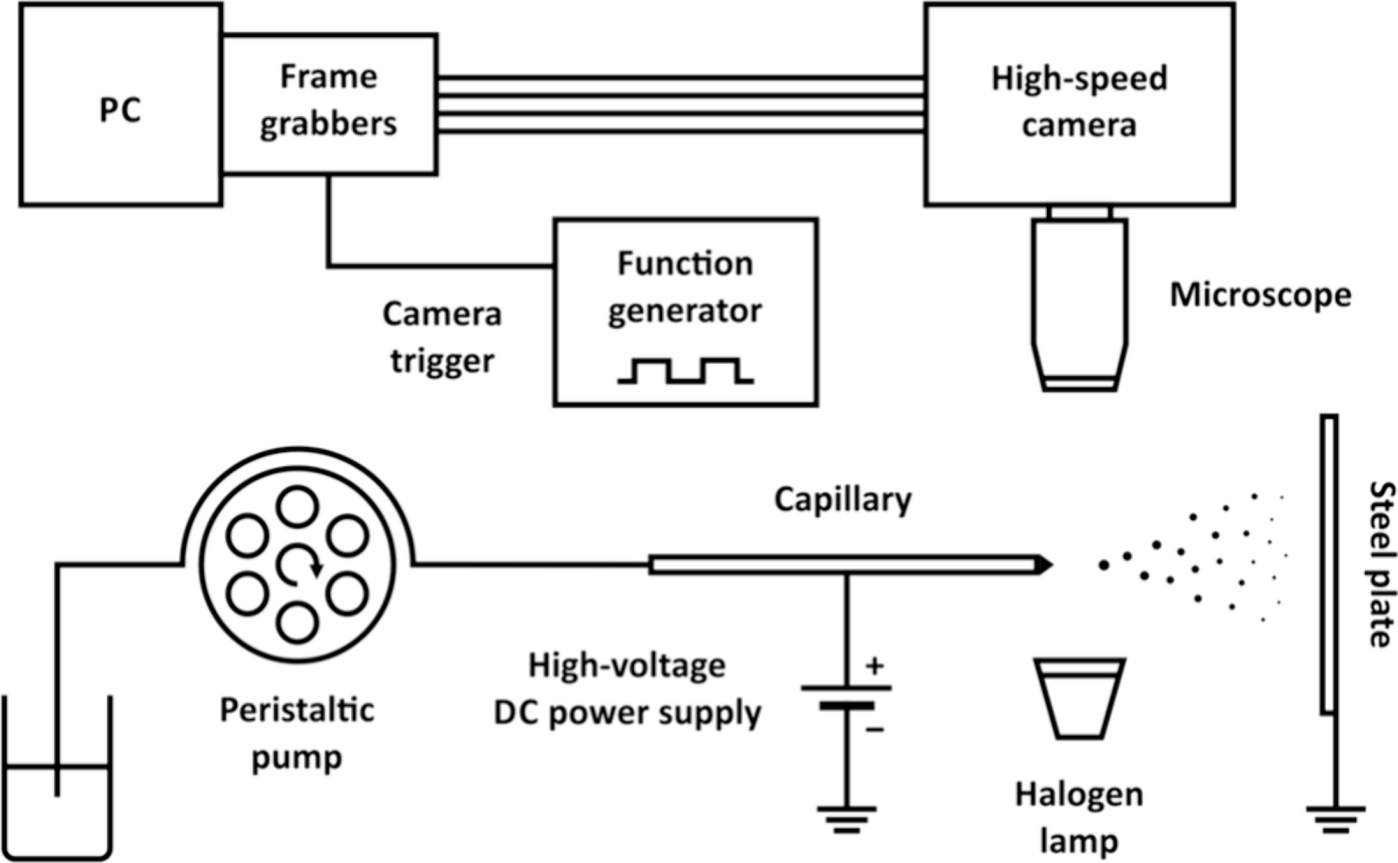
Experimental setup for high-speed imaging of microdroplets near the electrospray liquid meniscus.

To investigate the behavior of droplets near the liquid meniscus, a typical electrospray setup was built (Figure 1). Droplets were electrosprayed between two electrodes: an 82.5 mm long stainless steel ESI capillary (100 μm tip i.d., 270 μm tip o.d.; cat. no. 225-14915-00; Shimadzu, Kyoto, Japan) and a grounded counter electrode (stainless steel plate 8 × 8 cm). The capillary was positioned in a house-built 3D-printed case horizontally and perpendicularly to the plate with a distance between the tip and the counter electrode of ∼15 mm in each experiment. A positive 3.5 kV voltage was applied to the ESI capillary by a DC high-voltage power supply (MPS10P10/24/VCC; Spellman, Hauppauge, NY, USA). A peristaltic pump (PF102; Yotec Instruments, New Taipei City, Taiwan) was used to push the liquid sample to the capillary with the flow rate of 10 μL min^–1^ through a tubing system including a 455 mm long polyvinyl chloride pump tubing (250 μm i.d., 2 mm o.d.; cat. no. F116-0549-03; Pulse Instrumentation, Mequon, WI, USA), a 650 mm long fused-silica capillary (250 μm i.d., 350 μm o.d.; cat. no. 1010-36322; GL Sciences, Tokyo, Japan), and 42 mm long polytetrafluoroethylene tubing (300 μm i.d., 1.59 mm o.d.; cat. no. 58702; Supelco, Bellefonte, PA, USA).

### Imaging Setup

A high-speed camera (S710; Phantom, Wayne, NJ, USA), a microscope (SMZ745T; Nikon, Tokyo, Japan), and a light source consisting of a halogen lamp and an optical fiber bundle (OSL2IR; Thorlabs, Newton, NJ, USA) were used to record images of droplets near the capillary tip (Figure 1). The microscope was attached to the high-speed camera lens mount and fixed horizontally and orthogonally to the ESI capillary. As in brightfield microscopy, high-intensity white light was emitted from the light source and directed collinearly toward a microscope objective. The camera was triggered by a square wave signal from a function generator (5 V_pk_, 50 kHz; TFG-3605E; Twintex Instrument, New Taipei City, Taiwan). For acquiring and transferring images, two frame grabbers (Coaxlink Octo; Euresys, Liège, Belgium) were connected to a computer along with the high-speed camera. Within this study, the operating frame rate of the camera was 50 000 fps with a resolution 256 × 256 pixels and a 1 μs exposure time.

## Results and Discussion

**Figure 2:**
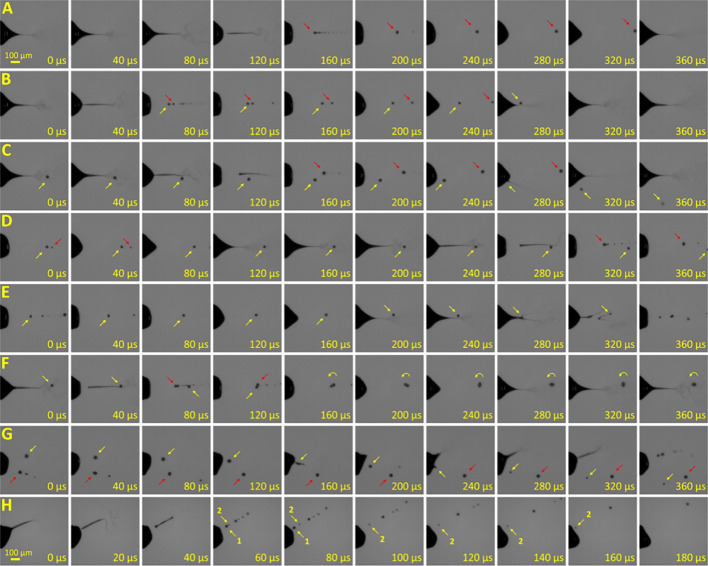
Consecutive images of the area close to the electrospray liquid meniscus obtained by the high-speed camera (see also Movie S1). The red arrows indicate droplets moving forward, the yellow arrows indicate droplets moving backward. (A) A droplet moves forward away from the meniscus. (B) A droplet first moves forward and then backward, then goes through the liquid meniscus and coalesces the liquid. (C) A droplet moves backward, collides with the meniscus and emits a spray of progeny droplets, then reduces in size and moves away from the meniscus. (D) A droplet decelerates, stops, and moves slowly forward again. (E) A droplet moves backward, collides with the next jet emission spindle, generating progeny sprays of smaller droplets, then reduces in size and moves forward. (F) A droplet moves backward, then merges with a microdroplet generated in the next jet emission spindle breakup, and the resulting droplet rotates. (G) After moving backward, a droplet bounces off the liquid meniscus twice in a row and moves forward. (H) Two droplets move backward and coalesce with the liquid in sequence. Panels A–F correspond to the MeOH–water (25:75, v/v) solution; panels G and H correspond to MeOH–water (25:75, v/v) with 0.5% ammonium hydroxide.

We noticed that, in the vicinity of the ESI emitter tip, a liquid spindle (or ligament) breakup generated a series of droplets in a pulsating cone-jet mode (*cf*. refs (15) and (21)). The majority of them moved steadily toward the counter electrode (as marked with red arrows in Figure 2A; *cf*. Movie S1). However, some of the newly formed droplets moved backward to the liquid meniscus (yellow arrows). In most cases, a returning droplet was the closest one to the emitter tip in a series of droplets (Figure 2B–G). Even so, we occasionally witnessed two or three droplets in a single emission being returned to the meniscus (Figure 2H). Following a change to reverse motion, returning droplets underwent various scenarios. It should be noted that complete coalescence with the liquid cone (Figure 2B), collision and bouncing off the meniscus with partial coalescence (Figure 2C), and noncoalescent bouncing (moving backward and stopping without reaching the meniscus, Figure 2D) were previously reported by Agostinho et al. for water.^20^ We observed additional variations, such as collision with the next jet emission spindle (Figure 2E); a droplet that bounces off the meniscus, generating a progeny jet emission, moves away from the meniscus, stops, then returns to the meniscus again and bounces off again, generating a progeny jet emission (double bounce; Figure 2G); and a droplet that collides and totally coalesces with a droplet from the next jet emission spindle, with rotation of a subsequent droplet (Figure 2F). This rotating droplet either moves toward or away from the meniscus—we witnessed both. All of the scenarios (A–H) are presented in a slow-motion video (Movie S1) to evidence the above-mentioned behaviors more clearly.

We studied methanol–water solution (25:75, v/v) as a typical solvent for ESI-MS without and with small amounts of three additives: ammonium acetate (10 mM), ammonium hydroxide (0.5%, v/v), and formic acid (0.5%, v/v). In MS, these solvent modifiers are commonly used to improve ESI performance.^3,22−24^ Thus, we had four types of electrosprayed liquids to examine. We recorded 20 000 frames for every type and reproduced the experiments over 4 days. The average number of returning droplets per 100 jet emissions is presented in Table 1. For a methanol–water solution without additives and a methanol–water solution with ammonium hydroxide, the numbers are 28 and 18 out of 100 jet emissions, respectively. Remarkably, the addition of formic acid and ammonium acetate decreased the number of returning droplets to approximately two droplets per 100 jet emissions.

**Table 1:** Statistical Analysis of Returning Droplets

condition	average number of returning droplets per 100 jet emissions (***n*** = 4)
MeOH–water (25:75, v/v)	28 ± 8
ammonium acetate (10 mM) in MeOH–water (25:75, v/v)	2 ± 3
formic acid (0.5%, v/v) in MeOH–water (25:75, v/v)	2 ± 1
ammonium hydroxide (0.5%, v/v) in MeOH–water (25:75, v/v)	18 ± 10

In ESI-MS, nanoscale droplets produce gas-phase ions that are detected by a mass analyzer, while the role of large microdroplets is lesser.^5,7,25−27^ In the present study, we observed that the majority of microscale droplets are produced by a spindle breakup in a pulsating cone-jet mode. It is likely that many of these microdroplets are wasted for MS. However, here we consider returning microscale droplets as spontaneously “recycled” for MS due to the above-mentioned scenarios, except for A and D (droplet stops, moves backward a little, and goes forward again), because the liquid in droplets moving backward either returns to the liquid cone or produces progeny jet emissions of nanoscale droplets. We estimated the percentage of liquid recycled due to retrograde motion phenomenon as ∼5% for methanol–water solutions. It is interesting that some of the recharged droplets immediately formed a secondary Taylor cone and emitted progeny droplets. This observation suggests that the amount of electric charge transferred to such a droplet is sufficient to surpass the Rayleigh limit. In fact, droplet charging was previously observed when microdroplets were exposed to the microenvironment of a corona discharge electrode.^28^

The presence of droplets moving backward to the emitter in electrospray can be explained by the fact that droplets carry both positive and negative ions.^29−31^ For instance, Maze et al. reported that, in positive electrospray, a small fraction (∼1%) of droplets that reach a detector are negatively charged due to bipolar fission processes and field-induced polarization.^29^ Gao and Austin investigated the mechanism of charge separation and droplet breakup using microparticles as probes.^31^ They found that ∼20% of particles carry charges opposite to the voltage applied to the ESI capillary. Zhou and Cook noted that electrophoretic separation of ions can lead to an axial charge gradient, and it can persist in the electrosprayed droplets at least until the first droplet fission.^32^ In the positive ESI mode, the surface of the liquid cone is positively charged due to the electrostatic repulsion.^33,34^ The liquid spindle—polarized by a strong electric field—breaks up and generates a series of negatively (closer to the meniscus) and positively (farther from the meniscus) charged droplets through varicose instabilities and fission processes.^14,15,20,29^ If the electric charge of a droplet is not enough for the electrostatic repulsion from the cone to the counter electrode, the droplet could be attracted back to the liquid meniscus due to the droplet’s negative net charge. We examined this behavior by adding different electrolytes into the solution (Table 1). Adding a small amount of formic acid decreases the number of returning droplets by lowering the pH of the solution, *i.e.*, increasing the concentration of H^+^ ions of the sample, including the portion present in electrosprayed droplets directly after the emission.^5,35^ Recently, Konermann et al. showed that adding ammonium acetate into the ESI solution provides moderate acidification of nascent electrosprayed droplets and facilitates the average ESI droplet pH drop of ∼1.6 units.^36^ In other words, ammonium acetate enhances the presence of mobile cations in droplets. Therefore, the number of the returning droplets decreases in the ammonium acetate case. In contrast, ammonium hydroxide raises the pH of the ESI solution, increasing the concentration of OH^–^ anions.^37−39^ Mansoori et al. showed that the pH of the ESI solution with ammonium hydroxide moderately decreases after spraying in the positive mode due to the evaporation of ammonia.^40^ However, in the current study, returning droplets were observed immediately after spraying, so evaporation processes were negligible for them. Therefore, we witnessed more returning droplets for the solution with ammonium hydroxide (more anions) than with ammonium acetate and formic acid (more cations). The present result is another evidence for charge separation in electrospray droplets induced by a strong electric field.

## Conclusion

In ESI-MS, nanodroplets are the main providers of the gas-phase ions. Microdroplets hardly impact the ESI-MS response. Here, we investigated the previously reported phenomenon of retrograde motion of microdroplets in electrospray using the electrolyte solutions that are frequently used in ESI-MS. The above observations of retrograde motion of charged droplets in electrospray were enabled by high-speed imaging. The phenomenon reveals the ongoing recycling of sample liquid in electrosprays. Such recycling certainly contributes to the sample utilization efficiency in electrospray ionization. We believe this finding further contributes to the holistic description of the ESI mechanism.

## Supporting Information Available

Video illustrating the returning microdroplets (MP4)
